# Effect of Bout Length on Gait Measures in People with and without Parkinson’s Disease during Daily Life

**DOI:** 10.3390/s20205769

**Published:** 2020-10-12

**Authors:** Vrutangkumar V. Shah, James McNames, Graham Harker, Martina Mancini, Patricia Carlson-Kuhta, John G. Nutt, Mahmoud El-Gohary, Carolin Curtze, Fay B. Horak

**Affiliations:** 1 Department of Neurology, Oregon Health & Science University, Portland, OR 97239, USA; harkerg@ohsu.edu (G.H.); mancinim@ohsu.edu (M.M.); carlsonp@ohsu.edu (P.C.-K.); nuttj@ohsu.edu (J.G.N.); horakf@ohsu.edu (F.B.H.); 2 Department of Electrical and Computer Engineering, Portland State University, Portland, OR 97207, USA; mcnames@pdx.edu; 3 APDM Wearable Technologies, Portland, OR 97201, USA; mahmoud.e@apdm.com; 4 Department of Biomechanics, University of Nebraska at Omaha, Omaha, NE 68182, USA; ccurtze@unomaha.edu

**Keywords:** mobility, Parkinson’s disease, bout length, wearable sensors, daily life

## Abstract

Although the use of wearable technology to characterize gait disorders in daily life is increasing, there is no consensus on which specific gait bout length should be used to characterize gait. Clinical trialists using daily life gait quality as study outcomes need to understand how gait bout length affects the sensitivity and specificity of measures to discriminate pathological gait as well as the reliability of gait measures across gait bout lengths. We investigated whether Parkinson’s disease (PD) affects how gait characteristics change as bout length changes, and how gait bout length affects the reliability and discriminative ability of gait measures to identify gait impairments in people with PD compared to neurotypical Old Adults (OA). We recruited 29 people with PD and 20 neurotypical OA of similar age for this study. Subjects wore 3 inertial sensors, one on each foot and one over the lumbar spine all day, for 7 days. To investigate which gait bout lengths should be included to extract gait measures, we determined the range of gait bout lengths available across all subjects. To investigate if the effect of bout length on each gait measure is similar or not between subjects with PD and OA, we used a growth curve analysis. For reliability and discriminative ability of each gait measure as a function of gait bout length, we used the intraclass correlation coefficient (ICC) and area under the curve (AUC), respectively. Ninety percent of subjects walked with a bout length of less than 53 strides during the week, and the majority (>50%) of gait bouts consisted of less than 12 strides. Although bout length affected all gait measures, the effects depended on the specific measure and sometimes differed for PD versus OA. Specifically, people with PD did not increase/decrease cadence and swing duration with bout length in the same way as OA. ICC and AUC characteristics tended to be larger for shorter than longer gait bouts. Our findings suggest that PD interferes with the scaling of cadence and swing duration with gait bout length. Whereas control subjects gradually increased cadence and decreased swing duration as bout length increased, participants with PD started with higher than normal cadence and shorter than normal stride duration for the smallest bouts, and cadence and stride duration changed little as bout length increased, so differences between PD and OA disappeared for the longer bout lengths. Gait measures extracted from shorter bouts are more common, more reliable, and more discriminative, suggesting that shorter gait bouts should be used to extract potential digital biomarkers for people with PD.

## 1. Introduction

Gait impairments are the leading cause of falls and disability in people with Parkinson’s disease (PD) [1,2,3,4]. Gait in people with PD is characterized by slow and short strides, shuffling, long double-support time, large stride-to-stride variability, and small arm swing [4,5,6]. Hence, quantitative gait assessment can be very useful for characterizing gait impairments [5], early detection of disease onset [7], tracking the progression of the disease [7], and testing the efficacy of interventions [8].

Recently, the use of wearable technology has made it feasible to quantify gait quality outside the laboratory during daily life [9,10,11,12,13,14,15,16,17,18,19,20,21,22,23,24,25,26,27,28,29]. Although the use of wearable technology is increasing in daily life environments, this is still a relatively new field of research in which algorithms and data analysis methods are under continuous development [11,20,21,30,31,32,33,34]. Furthermore, definitions about what is considered a gait bout (i.e., a period of continuous walking) differ, making comparisons across studies difficult [31,32,35,36,37,38,39].

It is also unclear what gait bout length to use to extract gait characteristics during daily life activities [11,34]. For example, gait bout lengths ranging from three steps to longer than 60 s have been used [11,25,34,40,41,42]. The advantage of shorter bouts is that the majority of walking during daily life occurs in shorter gait bouts, so gait measures can be averaged across more bouts [11,36,43]. However, many studies evaluating daily life gait quality characteristics have limited analysis to gait bouts longer than 10 s [41,44], 30 s only [34], 60 s only [11,21,45], or have used all gait bout lengths together [40,41]. The advantage of characterizing gait during longer bouts is that it focuses on what laboratory gait studies have called “steady-state gait” that is not affected by turns, gait initiation, gait termination, and hesitations that necessarily impact gait characteristics.

A few studies have shown that gait characteristics change as gait bout length increases [11,46,47,48]. Researchers and clinical trialists using daily life gait quality as study outcomes need to understand how gait bout length affects the sensitivity and specificity of measures to discriminate pathological gait, as well as the reliability of gait measures across gait bout length. It is possible that pathologies, like PD, that affect neural control of gait also affect how gait characteristics change as bout length changes. As PD impairs the ability to initiate and terminate muscle activity, even though peak and sustained muscle activity can eventually reach normal levels, we hypothesized that scaling of some specific gait measures may be impaired in PD. Thus, gait differences between PD and neurotypical subjects with similar age (Old Adults, OA) may be larger for short gait bouts because a greater percentage of time involved initiating and terminating muscle activity than for long gait bouts [49,50,51]. As a result, the discriminative ability of gait measures to separate PD from OA would be expected to be greater with shorter bout lengths. In addition, since more shorter than longer bout lengths are available for analysis during daily living, gait measures may also be more reliable for short than long gait bouts.

The aims of the study were to investigate: (1) the distribution and range of gait bout lengths available for analysis across a week of monitoring, (2) how gait bout length affects gait measures, and (3) how gait bout length affects reliability and discriminative ability of gait measures in people with PD and OA. We hypothesized that PD would affect the scaling of some gait measures with gait bout length and that shorter gait bout lengths (in contrast to larger gait bout length suggested in Reference [11]) should be used to extract gait measures in daily life for people with PD because of the better discriminative ability and reliability than longer gait bouts.

## 2. Methods

### 2.1. Participants

Twenty-nine people with idiopathic PD and 20 neurotypical adults of similar age participated in the study. Inclusion criteria for PD were a diagnosis of idiopathic Parkinson’s disease by a movement disorders neurologist with the United Kingdom Parkinson’s disease Society Brain Bank criteria, Hoehn and Yahr scores of II–IV, and complaints about gait disturbances. PD severity was assessed by a trained examiner using the Movement Disorders Society-Unified Parkinson’s Disease Rating Scale (MDS-UPDRS) [52]). Exclusion criteria for all subjects included the inability to follow protocol instructions, other factors affecting gait such as musculoskeletal disorders, uncorrected vision, or vestibular problems, or inability to stand or walk in the home without an assistive device. The experimental protocol was approved by the Institutional Review Board of the Oregon Health & Science University. All the participants provided informed written consent.

### 2.2. Daily Life Gait Data Collection

Subjects were asked to wear three inertial sensors (Opals by APDM, Inc., Portland, OR, USA): one on top of each foot, and one over the lower lumbar area with an elastic belt for a week of daily activities, for at least 8 h/day. The sampling rate of Opals was 128 Hz. The subjects removed the sensors at night to recharge the batteries. Data were stored in the internal memory of the Opals. After completion of a week of data collection, subjects returned sensors either by mail using a pre-paid box or to a research assistant study coordinator, who met the subject at their home. Data were then uploaded to a secure cloud-based database and downloaded to a local computer for further processing.

### 2.3. Measures of Gait

The gait algorithms used for extracting spatial and temporal measures were described previously [20,22], and were identical to that of Mobility Lab (APDM Wearable Technologies, Portland, Oregon), after the identification of gait bouts and turns [53,54,55]. Briefly, the algorithm first searches for possible bouts of gait from the feet data and for turns based on rotational orientation about the vertical axis of the pelvis. Second, individual steps were combined into potential bouts of gait, as long as the duration from one step to the next step is no longer than 2.5 s. Finally, each possible bout that contains at least 3 steps and is at least 3 s in duration is processed with the commercial gait analysis algorithms included in APDM Mobility Lab. The gait measures used for this analysis were gait speed, stride length, cadence, double-support, swing duration, and pitch of feet at initial ground contact. We purposefully chose not to use the coefficient of variation measures as the number of strides in the majority of bouts was less than the minimum number of strides required to accurately calculate a coefficient of variation (20 strides) [56,57].

We define gait bout length in terms of the number of strides in the bout because it helps to eliminate the differential effects of gait speed and stride length between groups on the gait bout length. Other studies have used gait bout duration [11,34,46], the distance traveled during a particular walk test [58], and the number of strides in a bout [42,47] as gait bout length measures.

### 2.4. Statistical Analysis

The Shapiro–Wilk test was used to assess the normality of variables. Since not all variables were normally distributed, a non-parametric test (specifically, Mann–Whitney U tests) was used throughout to investigate differences between PD and OA groups.

To investigate what bout length could be used to extract gait measures between groups, we investigated the range of gait bout lengths that are present in most of the PD and OA subjects. Specifically, we divided weekly gait bout lengths into 40 bins with empirical bin sizes of 5 strides, starting from 3 strides in a bout (e.g., 3–7, 8–12, 13–17,…, 203–207 bins) and calculated the percentage of subjects walking as a function of bout length for each group during a week of recording. We also calculated the percentage of bouts available as a function of bout length in each group. Bouts greater than 207 strides were discarded as only a very small percentage (0.9%) of gait bouts above this length were available across all the subjects during a week of daily recording.

To investigate the effect of bout length on gait measures, we restricted the rest of the analysis to bout bins in which at least 90% of subjects walked in both groups (i.e., OA and PD). We first visually compared how the gait metrics changed with bout length for the PD and OA groups, by plotting the mean and standard error of each gait metric as a function of the bout length. To statistically compare the effects of bout length on gait measures between the PD and the OA groups, we used growth curve analysis [59]. The overall curve for each gait measure was modeled with second-order (quadratic) orthogonal polynomials and fixed effects of group on all bin sizes for the gait bout length (for the growth model example, see Equations (1) and (2)). The OA group was treated as the baseline, and parameters were estimated for the PD group. The model also included random effects of participants on each bout length bin size. The fixed effects of the group (PD and OA) were added individually, and their effects on model fit were evaluated using the likelihood ratio test for model comparisons. Specifically, we started the analysis with a base model (model 1) that just has the Level 1 structure and the random effects, without any effects of group, then we added the fixed effect of group on the intercept (model 2), linear (model 3) term/component of the bout length, and finally, the full model with effects of group on all terms/components (i.e., intercept, linear, and quadratic) of the bout length (model 4). Improvements in model fit were evaluated using −2 times the change in log-likelihood, which is distributed as chi-square (χ^2^) with degrees of freedom equal to the number of parameters added.

Examples of the growth curve model for each gait measure are as follows:

(1) $$Level\;1:Y_{ij}=\beta_{0i}+(\beta_{1i}*X_{j})+(\beta_{2i}*{X_{j}}^{2})+\varepsilon_{ij}$$

(2) $$Level\;2:\{\begin{array}{c} \beta_{0i}=\gamma_{00}+(\gamma_{0Group}*Group)+\varphi_{0i}\\ \beta_{1i}=\gamma_{10}+(\gamma_{1Group}*Group)+\varphi_{1i}\\ \beta_{2i}=\gamma_{20}+(\gamma_{2Group}*Group)+\varphi_{2i} \end{array}$$

where, $\beta_{0i}$ is the intercept, $\beta_{1i}$ is the linear slope, and $\beta_{2i}$ is the rate of change of the quadratic curvature. Y is gait measure (example, gait speed), and X is a quadratic orthogonal polynomial of bout length bins. The fixed effects $\gamma_{0Group}$, $\gamma_{1Group}$, and $\gamma_{2Group}$ capture the systematic differences between groups (example, OA versus PD). The random effects $\varphi_{0i},\;\varphi_{1i},$ and $\varphi_{2i}$ capture (random) individual variability among participants.

To investigate the inter-day reliability of each gait measure and for each bout size bin, we used the intraclass correlation coefficient (ICC) [60] for daily averages. We only included days that had at least 3 values for each bout size range. To investigate the discriminative ability of each gait measure, we computed the receiving operating characteristics (ROC) curve and calculated the area under the curve (AUC) [61]. All statistical analyses were performed using R version 3.0.2, and Python version 3.6 software. The statistical significance was set to *p* < 0.05.

## 3. Results

### 3.1. Group Characteristics and Adherence

Participants with PD were mildly to moderately affected: MDS-UPDRS Part III score in the ON medication state: 34.66 ± 11.02, and Hoehn and Yahr stage: 2.07 ± 0.45 with I (*n* = 1), II (26), III (1), and IV (1). Table 1 shows the demographics of subjects who participated in this study. Age, height, and weight were similar between the PD and OA group. Adherence to the weekly recordings averaged 67.66 ± 12.53 h for the PD group and 64.67 ± 10.13 h for the OA group.

### 3.2. Frequency of Gait Bout Lengths over a Week of Daily Life

**Figure 1:**
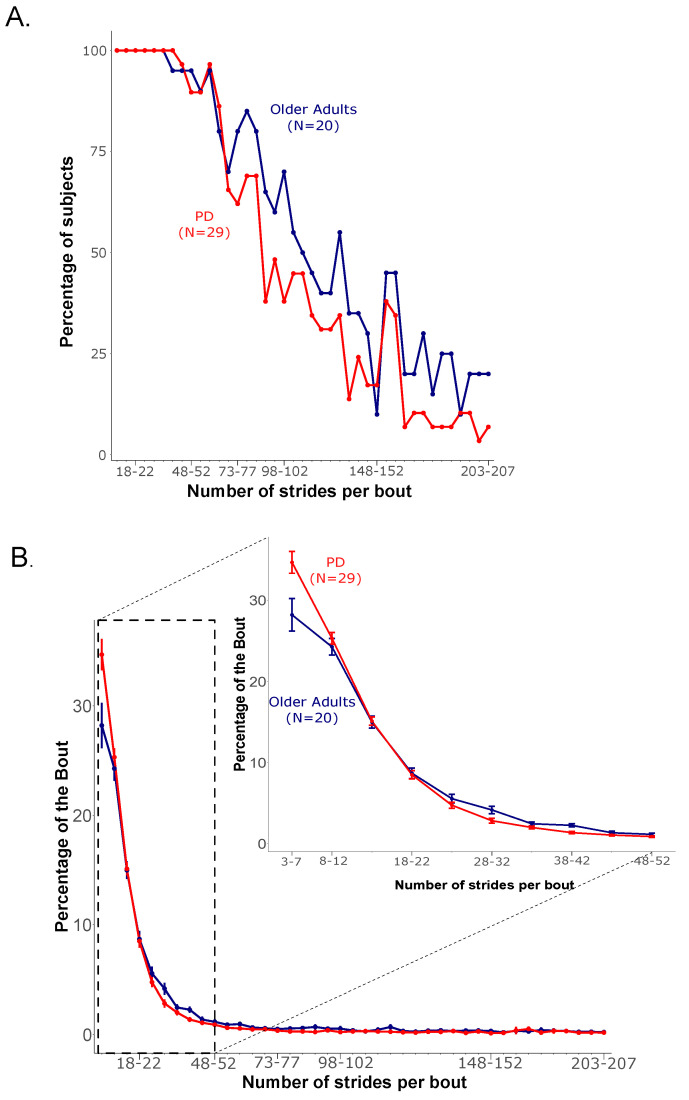
(**A**) Percentage of subjects walking as a function of bout length, and (**B**) percentage of all bouts performed by each participant.

The majority of participants in both groups walked with gait bouts of less than 53 strides per bout across the 7 days (Figure 1). Specifically, 90% of the OA group had less than 53–57 strides per bout, with the number of subjects dropping to 80% for less than 58–62 strides per bout. Similarly, 90% of the PD group had less than 53–57 strides per bout, with the number of subjects dropping to 86% for less than 58–62 strides per bout. Figure 1A shows the percentage of subjects (who had one or more bouts in a specific bin of bout length across the 7 days) as a function of gait bout length recorded in each group. Based on the above results, we chose to restrict the analysis to bouts with less than 53 strides for the rest of the analysis.

The majority of gait bouts (>50%) were very short bouts (less than 12 strides per bout) in 60% of PD and 52% in OA groups. The percentage of gait bouts follow a decreasing trend in each group as bout length increases. Further, the PD group had a larger percentage of bouts in the shortest bout (3–7 strides) length, compared to the OA group (35% versus 28%, respectively). Figure 1B shows the percentage of gait bouts available as a function of bout length in each group.

### 3.3. Gait Measures as a Function of Gait Bout Length

**Figure 2:**
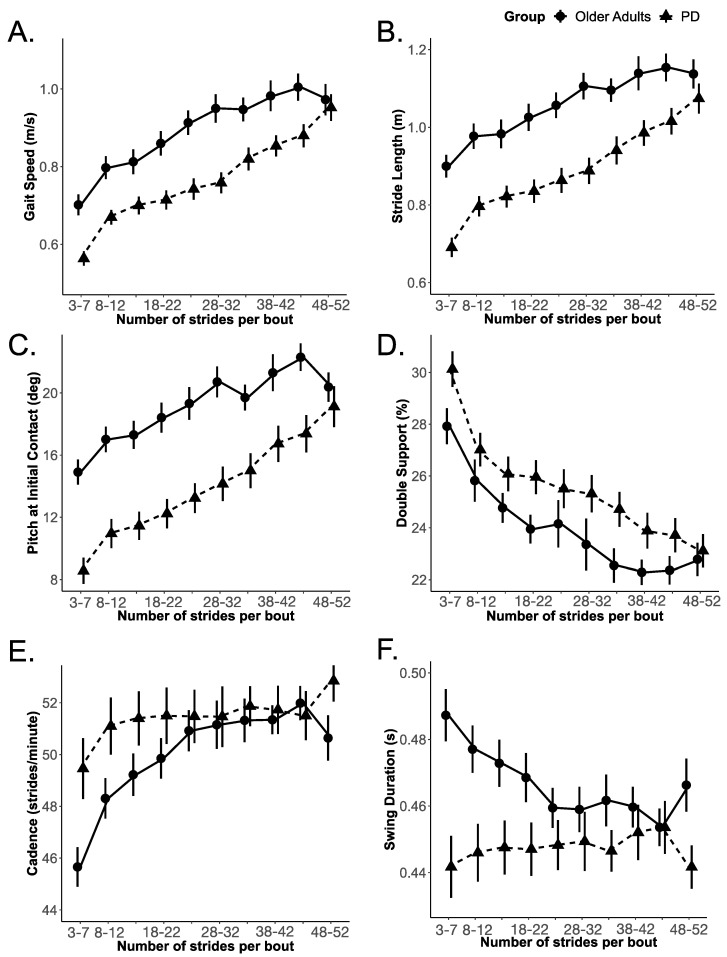
Gait measures (with a mean and standard error of the mean) as a function of bout length for OA and PD; (**A**) Gait speed, (**B**) Stride length, (**C**) Pitch at initial contact, (**D**) Double support, (**E**) Cadence, and (**F**) Swing duration.

Gait speed, stride length, and foot pitch at initial contact increased, and double-support time decreased with increasing gait bout length in both the OA and PD groups. These gait measures appeared to change similarly with bout length for both groups. However, unlike these four gait metrics, cadence and swing duration did not scale with bout length for the PD group, whereas the OA group did scale with bout length (Figure 2).

**Figure 3:**
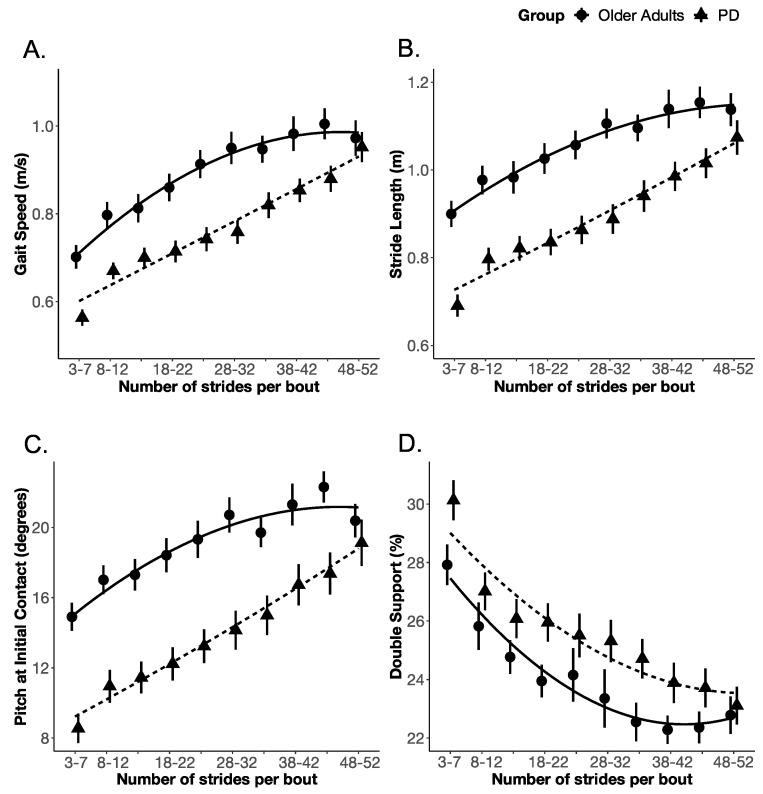
The growth curve analysis (PD vs. OA) model fit for (**A**) Gait speed, (**B**) Stride length, (**C**) Pitch at initial contact, and (**D**) Double-support.

**Figure 4:**
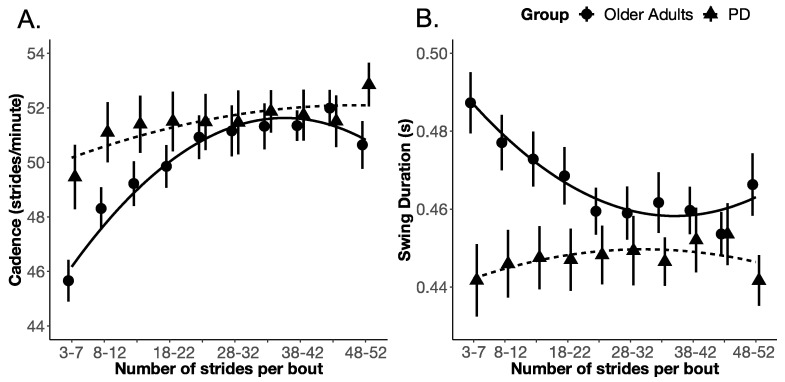
The growth curve analysis (PD vs. OA) model fit for (**A**) cadence and (**B**) Swing duration.

The PD group significantly differed from the OA group in both the slope and rate of change (but not the intercept) of cadence and swing duration with bout length. None of the other gait measures (i.e., gait speed, stride length, the pitch of foot at initial contact, and double-support time) showed the same effect as that of cadence and swing duration, as shown in Table 2. Specifically, for cadence, the effect of the group on the intercept (i.e., model 1 to model 2) did not improve model fit (χ^2^ (1) = 1.132; *p* = 0.287), however, the effect of the group on the linear (i.e., model 2 to model 3) and quadratic (i.e., model 3 to model 4) terms improved the model fit (χ^2^ (1) = 5.091; *p* = 0.024, and χ^2^ (1) = 9.081; *p* = 0.003, respectively), indicating that the OA and PD groups differed in the slope and rate of change of cadence. Similarly, for swing duration, the effect of the group on the intercept did not improve model fit (χ^2^ (1) = 1.677; *p* = 0.195); however, the effect of the group on the linear (χ^2^ (1) = 7.294; *p* = 0.007) and quadratic terms did improve model fit (χ^2^ (1) = 15.547; *p* = 0.000), indicating that the OA and PD groups differed in the slope and rate of change of swing duration. The details of the fixed effect parameter estimates, and their standard error along with *p*-values estimated using the normal approximation for the *t*-values, are summarized in Appendix A, Table A1. The growth curve model fit for each gait measure is shown in Figure 3 and Figure 4.

### 3.4. ICC and AUC of Gait Measures as a Function of Gait Bout Length

**Figure 5:**
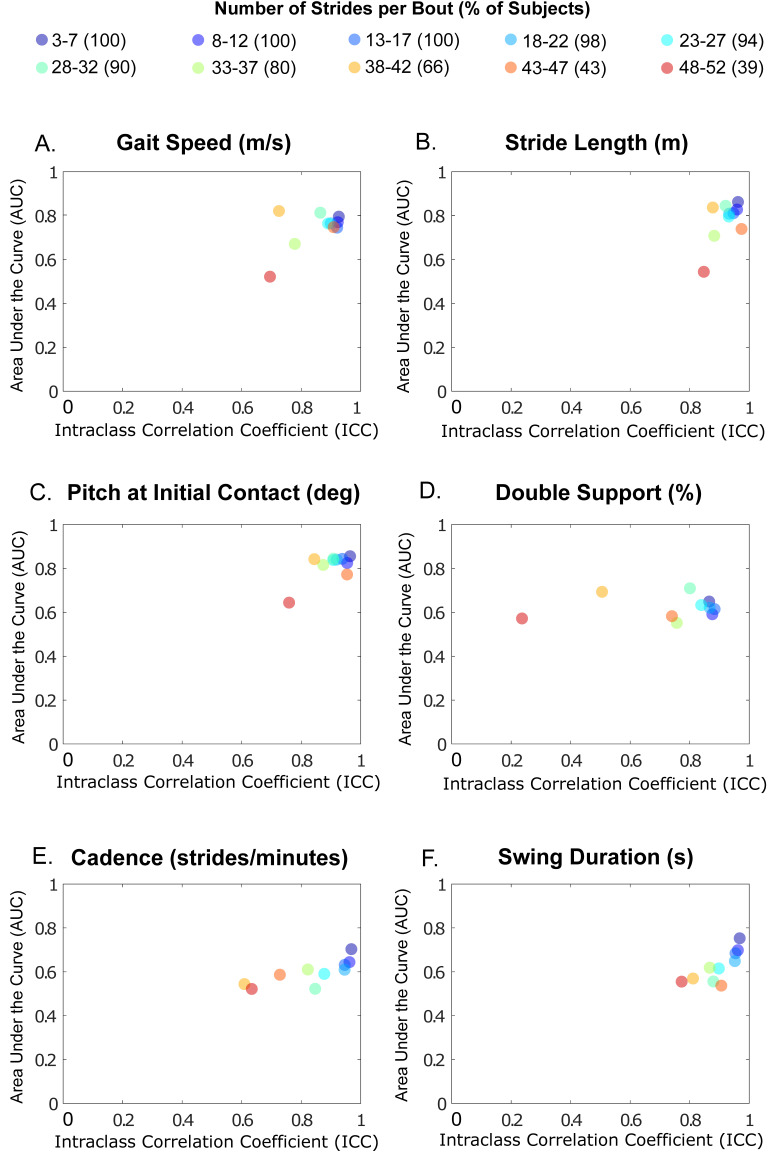
The intraclass correlation coefficient (ICC) versus area under the curve (AUC) of (**A**) Gait speed, (**B**) Stride length, (**C**) Pitch at initial contact, (**D**) Double support, (**E**) Cadence, and (**F**) swing duration for various bout lengths. The total percentage of participants are shown inside the bracket of bout length bins.

The ICC and AUC characteristics tended to be greater for shorter bout lengths for each gait measure, as shown in Figure 5. Furthermore, ICC and AUC characteristics differed, depending upon the specific gait measure, and generally tended to decrease as gait bout length increased. For example, ICC and AUC for gait speed, stride length, and pitch of the foot at initial contact were tightly clustered around the top right corner, except for the longest bouts, in which ICCs were smaller. Cadence and double-support time showed gradually smaller AUCs for the longer gait bouts.

## 4. Discussion

Most gait bouts are quite short during daily life. In fact, our study showed that 60% of gait bouts in PD and 52% of gait bouts in OA consist of very short bouts (3–12 strides), and at least 75% of bouts were less than 22 strides (76% in OA and 83% in PD). Very short gait bouts in daily life agree with the results of previous studies showing that 55% of gait bouts in OA and 59% of gait bouts in PD were less than 10 s [11]. Another study in young neurotypical adults found that 40% of gait bouts were less than 6 strides, and 75% of gait bouts were less than 20 strides [36]. Likewise, 50% of gait bouts were less than 10 strides in the frail elderly [47], 50% of bouts were less than 13 s [43], and at least 50% of bouts were less than 8 s in both faller and non-faller groups [62]. Such a preponderance of short walking bouts in daily life likely reflects natural mobility to accomplish daily tasks as well as natural mobility during daily activities and moving in constrained environments, such as a house.

Gait speed and stride length scaled up with increased gait bout length for both the OA and PD groups. This result agrees with a previous study in PD and OA groups [11]. Since the inertial sensors were on the feet, we also observed that the foot pitch angle was more dorsiflexed the longer the bouts (and faster gait with longer steps). Not surprisingly, as gait speed increased with bout length, double-support time decreased [63]. Thus, PD does not interfere with the scaling up of the pace of gait as bout length increases.

Unlike these pace measures, the temporal gait measures of cadence (strides per minute) and swing time duration scaled differently with longer bout lengths in the PD group alone. The result of the lack of change in cadence across bout lengths for PD, but significant changes for OA, is that cadence was higher than OA in shorter bouts but within the normal range for longer bouts. Similarly, the PD group swing time was shorter than OA for shorter bouts but within the normal range at longer bouts. Thus, differences between groups were most prominent for shorter bouts but were absent with longer bout length.

Swing duration showed a similar trend to not scale up with gait bout length in the PD group, unlike the OA group that had shorter swing durations the longer (and faster) subjects walked. Swing duration in the PD group was shorter than OA for the shortest gait bouts (3–7 strides) but then tended to be longer than normal as gait bouts lengthened, primarily because healthy OA subjects quickly shortened swing duration as bout length (and therefore gait speed) increased. The inability to change cadence with bout length likely resulted in unchanged swing duration.

It is unclear why cadence is higher than normal for shorter bout lengths and did not increase with bout length in the PD group. Similar to our results, a previous study also showed that increasing treadmill speed resulted in an increase in both cadence and stride length in the OA group but only stride length in a PD group [64]. High cadence has been associated with PD previously and thought to represent partial compensation for short stride length due to weak (bradykinetic) plantar flexion torques that should propel subjects forward for gait [64].

Inability to scale up temporal vigor of gait (steps per minute with faster swings) when intending a longer walk may be unique to PD and reflected in their bradykinetic gait. Scaling up the vigor of motor output has been attributed to control by the basal ganglia [51]. The loss of dopamine neurotransmitter results in reduced basal ganglia output to other motor centers, including the thalamus to motor cortex, the brainstem, and cerebellar locomotor centers [3]. People with PD have previously been shown to lack scaling up postural forces in responses to longer, more destabilizing surface translations like younger and older neurotypical adults [65].

Our ICC and AUC results suggested that the shorter bouts yield the most reliable and discriminative gait measures to characterize parkinsonian gait compared to age-matched controls. It is unclear why bout-to-bout reliability was better for shorter bouts than for longer bouts, especially for cadence and double-support time. There was a disparity in the number of gait bouts between shorter vs. longer gait bouts, but that should not necessarily result in better ICCs for shorter bouts. Perhaps longer gait bouts, associated with prescribed aerobic exercise as subjects walked around outdoors, resulted in a greater variety of voluntary (less automatic) gait temporal features than shorter gait bouts. Shorter bouts are also less likely interrupted by turns and obstacles that would alter temporal components of gait compared to longer bouts. Nevertheless, the goal to measure what has been called in laboratories as “steady-state gait” without effects of gait initiation, gait termination, turns, and other transitions may not be a feasible or desirable goal when measuring gait characteristics in natural daily-life activities.

Our AUC results are inconsistent with a previous study in which the authors found that gait bout length between 30 and 60 s to gait bout length > 120 s showed more discriminative power to discriminate people with PD from age-matched healthy control subjects than short bouts [11]. Also, the authors found that none of the gait measures discriminated gait characteristics in people with PD from healthy age-matched control subjects for very short bouts (gait bout length < 10 s). This inconsistency in the results may be partly due to the extensive validation criteria we use to ensure that the periods of gait analyzed are during periods of steady-state gait and straight progression. The earlier study used accelerometers placed on the lower back to detect strides with a different methodology. In particular, our analysis excluded gait during turns, which cannot be detected reliably without the use of both an accelerometer and a gyroscope. This is the first study providing evidence for the importance of shorter bouts as a robust marker of PD gait.

There are several limitations to the current study. First, we had a modest sample size. Future work with larger cohorts is needed to investigate if these findings would generalize. Second, our shorter and more reliable gait bouts may be in line with the moderate to long gait bouts detected with accelerometers on the lower back [11], because we require at least two consecutive left-right-left-right steps, and excludes step initiation or turns that may be included in the gait bouts detected with lower back accelerometry. Third, we had three sensors (two on the feet and one over the lumbar), and we asked participants to charge sensors at night while other approaches have only one sensor incorporating accelerometer at the lumbar for a week of recording. Finally, we did not consider the effect of gait speed and motor fluctuations while exploring the effect of gait bout length on gait measures. It is possible that people with PD walk faster and take longer gait bouts in the ON medication state. Hence, future work should also explore the effect of bout length, with gait speed and medication state as covariates.

## 5. Conclusions

Our findings show the potential of focusing gait analysis on the shortest walking bouts in daily life to develop digital biomarkers for people with PD. Swing duration during short walking bouts over 7 days of monitoring provided the most sensitive and specific, and most reliable gait measure of parkinsonian gait. The inability of people with PD to scale up some of their temporal characteristics of gait with an increase of gait bout length may be unique to this particular type of neurological disease and requires further study.

## Figures and Tables

**Table 1:** Demographics and activity characteristics of each group.

	OA (N = 20)		PD (N = 29)		*p*
	Mean	SD	Mean	SD	
Age (years)	66.85	7.16	67.66	5.27	0.45
Height (m)	1.70	0.18	1.71	0.13	0.32
Weight (kg)	74.61	9.38	75.95	12.74	0.91
Male/Female (#)	12/8		17/12		
Bouts/hour (#)	22.37	1.95	20.72	1.30	0.68
Turns/hour (#)	102.89	10.96	77.67	7.59	0.03

OA = Old Adults; PD = Parkinson’s disease; SD = standard deviation.

**Table 2:** Results of growth curve analysis with second-order (quadratic) orthogonal polynomial of bout length with the fixed effect of group (OA and PD) for each gait measure.

Gait Measure	Term/Component of Bout Length	Goodness of Fit (Log Likelihood)	χ^2^ (1)	*p*
Gait Speed (meters/second)	Intercept	418.596	11.015	0.001
	Linear	419.489	1.785	0.182
	Quadratic	423.150	7.323	0.007
Stride Length (meters)	Intercept	432.732	13.695	<0.001
	Linear	436.061	6.656	0.010
	Quadratic	438.011	3.901	0.048
Pitch at Initial Contact (degrees)	Intercept	−1184.259	14.521	<0.001
	Linear	−1180.753	7.012	0.008
	Quadratic	−1178.601	4.304	0.038
Double Support (%)	Intercept	−1061.506	0.975	0.324
	Linear	−1059.899	3.214	0.073
	Quadratic	−1059.399	0.999	0.317
Cadence (strides/minute)	Intercept	−1106.360	1.132	0.287
	Linear	−1103.814	5.091	0.024
	Quadratic	−1099.274	9.081	0.003
Swing Duration (seconds)	Intercept	1206.537	1.677	0.195
	Linear	1210.184	7.294	0.007
	Quadratic	1217.958	15.547	<0.001

## Appendix A

**Table A1:** Parameter estimates for growth curve analysis of effect of group on gait measures in OA and PD groups.

Gait Measure	Term/Component of Bout Length	Estimates	Std. Error	*t*	*p*
Gait Speed (meters/second)	Intercept	0.892	0.025	35.549	0.000
	Linear	0.271	0.028	9.629	0.000
	Quadratic	−0.089	0.024	−3.701	0.000
	PD: Intercept	−0.128	0.033	−3.916	0.000
	PD: Linear	0.058	0.037	1.570	0.116
	PD: Quadratic	0.088	0.031	2.808	0.005
Stride Length (meters)	Intercept	1.056	0.031	34.286	0.000
	Linear	0.237	0.026	9.111	0.000
	Quadratic	−0.059	0.023	−2.559	0.010
	PD: Intercept	−0.166	0.040	−4.144	0.000
	PD: Linear	0.095	0.034	2.790	0.005
	PD: Quadratic	0.061	0.030	2.014	0.044
Pitch at Initial Contact (degrees)	Intercept	−19.025	0.958	−19.857	0.000
	Linear	−5.829	0.909	−6.412	0.000
	Quadratic	1.915	0.743	2.577	0.010
	PD: Intercept	5.157	1.246	4.140	0.000
	PD: Linear	−3.744	1.189	−3.148	0.002
	PD: Quadratic	−2.059	0.974	−2.115	0.034
Double Support (%)	Intercept	24.097	0.651	37.002	0.000
	Linear	−4.585	0.478	−9.586	0.000
	Quadratic	1.969	0.563	3.500	0.000
	PD: Intercept	1.441	0.847	1.702	0.089
	PD: Linear	−1.046	0.626	−1.670	0.095
	PD: Quadratic	−0.739	0.735	−1.006	0.314
Cadence (strides/minute)	Intercept	49.988	0.914	54.719	0.000
	Linear	4.596	0.927	4.959	0.000
	Quadratic	−2.882	0.577	−4.994	0.000
	PD: Intercept	1.440	1.188	1.212	0.225
	PD: Linear	−2.608	1.209	−2.158	0.031
	PD: Quadratic	2.412	0.756	3.191	0.001
Swing Duration (seconds)	Intercept	0.467	0.007	62.463	0.000
	Linear	−0.022	0.008	−2.966	0.003
	Quadratic	0.016	0.004	4.205	0.000
	PD: Intercept	−0.020	0.010	−2.039	0.041
	PD: Linear	0.027	0.010	2.706	0.007
	PD: Quadratic	−0.022	0.005	−4.387	0.000
